# Factors affecting the emotional reactions of patient relatives who receive news of death: a prospective observational study

**DOI:** 10.1186/s40359-022-00763-2

**Published:** 2022-03-08

**Authors:** Bülent Barış Güven, Özgür Maden, Ayşe Dudu Satar, Ayşın Ersoy

**Affiliations:** 1 Department of Anesthesia and Reanimation, Sultan 2. Abdulhamid Han Training and Research Hospital, University of Health Sciences Turkey, P.O. Box: 34668, Üsküdar, Istanbul, Turkey; 2 Department of Psychiatry, Sultan 2. Abdulhamid Han Training and Research Hospital, University of Health Sciences Turkey, P.O. Box: 34668, Üsküdar, Istanbul, Turkey; 3 Department of Nursing Science, Sultan 2. Abdulhamid Han Training and Research Hospital, University of Health Sciences Turkey, P.O. Box: 34668, Üsküdar, Istanbul, Turkey

**Keywords:** Intensive care, Death, Notification, Grief reaction, Breaking bad news

## Abstract

**Background:**

Reporting the death of relatives to a family member is a very stressful task for physicians. Grief reactions differ from person to person.

**Methods:**

Demographic data of 100 patients who died after staying in ICU for more than three days were recorded. For each patient, one of the family members filled a form which contained their own age, gender, education level, marital status, number of children, degree of relationship, psychiatric treatment status, living in the same house as the patient, and whether they had ever visited the ICU before. Grief reactions were evaluated in five different categories: normal grief response, initial shock reaction, denial, feeling guilty and anger.

**Results:**

When the death was reported, 55.0% of the relatives accepted this situation as normal, 19.0% felt guilty and 14.0% showed an initial shock reaction. The results showed that for a one-unit increase in the patient's age, the probability of the denial reaction among relatives was reduced by 746 times and the probability of feeling guilty was reduced by 698 times.

**Conclusion:**

The rate of denial and guilt in the grief reactions among patient relatives when given news of death in the intensive care unit increases with the decrease in patient age.

## Background

Human beings feel the need to establish relationships based on love and trust throughout their lives. When they are happy or sad, they want to be with the people with whom they have formed these deep relationships. However, when these loved and trusted people are lost, grief reactions occur in which emotions such as anxiety, sorrow, and sadness are experienced intensely. “Mourning” for the person who was lost is a natural process that must be experienced. During the grieving process, surviving individuals protest the loss emotionally and may experience some mental problems [1]. In order for one to continue life in a normal and healthy way, it is necessary to mourn and complete the grieving process. At the end of this grieving process, one learns to live with the loss by making it a part of life. Grieving does not mean forgetting about the deceased or not loving them anymore. It simply means accepting loss and the feelings associated with it and learning to cope and living with those feelings. In other words, it is to be able to psychologically bury the person who was physically buried in the grave. When we observe many grieving people, we also notice many different grieving reactions. Some individuals show very severe grief reactions, while others show mild grief reactions. While some show grief reactions as soon as they receive news of the loss, others postpone their grief and experience it later on. All these differences show that we are different from each other personally and we experience grief by reacting differently.

Grief reactions have a beginning, phases, and an end. In the literature, the grieving process was defined differently by each researcher. E. Kübler-Ross defined the grieving process in five stages: denial, anger, bargaining, depression and acceptance. According to the Bowlby and Parkes model, the grieving process consists of shock, searching, disorganization and rebuilding stages [2, 3]. However, these stages of the grieving process can change or differ within themselves. Today, it is accepted that the grieving process is not linear. The idea that “grief is very painful in the beginning; this pain decreases and feels better with time” is no longer valid. The accepted view today is that grief is a chaotic and circular process [4].

Feedback from grieving family members shows that both good communication and the completeness and accuracy of information about patients play important roles in the grieving process [5, 6]. Therefore, at this stage, an empathetic approach and communication with the people who first convey the news of death are important. It is difficult to tell a family member that their relative has died. Physicians undertake this difficult task, as deaths occur most frequently in hospitals. However, in most countries, including Turkey, this subject is not included in the medical school education system. Benenson et al. [7] reported that only 12% of medical schools in the United States had education about death reporting in their curriculum. For this reason, each physician decides how to give this news in line with their own experience, character and mood. It is more difficult for physicians to give news of death and for patient relatives to accept this news especially in sudden and unexpected deaths in emergency services. In any case, the reaction to the news of a death can be intense and cause emotional outbursts. Behavioral reactions to news of death depends on many characteristics such as the level of relationship, the cause of death, the educational level of the patient’s relative, marital status, age and sociocultural structure. They can express their sadness in different ways, from silence to crying, screaming to various bodily movements. During these behaviors, health workers may also be exposed to emotional and physical violence. Knowing the factors that may cause these emotional and physical reactions among relatives of the patients will positively contribute to the relationship between the physician and the patient’s relative.

## Theoretical framework

The grieving process is expressed as an individual and unique process. There is no comprehensive theory that explains a normal grieving process. Studies show that grief consists of a series of phases and stages with predictable symptoms that change over time, and these stages do not develop in the same order or in the same way [1]. However, theoretically, with the news of death, the first phase of the grieving process begins. Approximately 60% of deaths occur in hospitals and the majority of hospital deaths occur in intensive care units [8]. Therefore, we can state that some of the factors affecting the grieving process are associated with the intensive care period. In the literature, there is no study evaluating the reactions of relatives and the factors which affecting them in intensive care units, where news of death is most frequently reported. In our study, based on this idea, we aimed to highlight the factors which affect the emotional reactions of relatives of the patients being monitored and treated in the intensive care unit (ICU). Thus, by examining the factors affecting the first phase of grief, awareness will be raised for health care professionals to help people complete the grieving process in a healthy manner.

## Method

The study was performed in the advanced intensive care units with a total of 36 beds of the Health Sciences University, Sultan 2. Abdülhamid Han Training and Research Hospital, Anesthesiology and Reanimation Clinic, between May 2019 and November 2020, after the approval of the ethics committee. Voluntary consent was obtained from the relatives of patients to confirm that they wanted to participate in the study. Patients who were monitored in the intensive care unit for longer than 72 h with Acute Physiology and Chronic Health Evaluation II (APACHE II) score above 25 or expected death rate higher than 50% were included in the study. Among patient relatives (PR), the person who visited the most and received regular information was selected and included in the study. Patients with an APACHE II score of less than 25 or an expected death rate of less than 50%, a hospital stays of less than 72 h in the ICU, currently receiving psychological medication, and relatives who did not agree to participate in the study were excluded from the study. The study was terminated when the number of deaths reported reached 100. In this study the diagnosis, age, gender, length of stay in the ICU, APACHE II scores and Glasgow coma scores (GCS) of the patients were recorded. Patients were divided into diagnostic classes according to their medical conditions and histories at the time of admission. In the interviews, the relatives of the patients filled the sociodemographic data form prepared by the researchers which included information about age, gender, education level, marital status, number of children, degree of closeness, psychiatric treatment status, living in the same house as the patient, and whether they have ever visited the ICU before. Finally, when a face-to-face death notification was made, the first grief reactions of the relatives of the patients were recorded in the patient relative data form. All death reports were made by three different intensive care specialists who were familiar with the study protocol. The grief reactions of the patients were evaluated in five different categories (normal, initial shock reaction, denial, guilt and anger), as stated by Naik [9].

### Statistical analysis

Statistical analysis was performed using the SPSS (ver.22.0, Chicago, II, USA) program. The recorded data were transferred to the computer. Frequency, percentage, mean value and standard deviation were used to define the data. First of all, the Kolmogorov Smirnov test was used to examine whether the data conformed to normal distribution or not. After this, the decision was made to examine the scale scores with parametric tests. Relationships between variables were analyzed by Pearson and Spearman correlation analyses. The correlations of the subgroups of the dependent variable with the independent variables was determined by multinomial logistic regression (MNLR) analysis. In statistical interpretations, 95% confidence interval values and *p* < 0.05 were considered significant.

## Results

Data obtained from the participants were evaluated within the scope of descriptive, relational and MNLR analyses. The mean age of the patients participating in the study was 70.15 ± 14.88 years, and the mean age of PR was 48.71 ± 12.35 years. Of PR, 60.0% were female, 38.0% were high school graduates, 78.0% were married, and 38.0% had 1–2 children. In terms of closeness, the majority of PR were daughters of the patients (42%). Of the PR, 51% had not visited any ICU before, and 37.0% of PR lived in the same house as the patient (Table 1). Of the patients, 54.0% were female, 30.0% had oncological disease, 22.0% had cardiovascular disease, and 20.0% had neurological disease. The length of stay was 17.96 ± 14.52 days. Their GCS was 6.89 ± 4.56, and their APACHE II score was 75.95 ± 12.59 (Table 2). When the death notification was made, 55.0% of PR met this situation normally, 19.0% felt guilty, and 14.0% showed an initial shock reaction. Denial (8%) and anger (4%) were observed less frequently (Table 3).

**Table 1:** Sociodemographic characteristics of relatives of inpatients in intensive care unit

	Frequency (n)	Percentage (%)
Gender of patient relatives		
Female	60	60.0
Male	40	40.0
Education level of the patient's relative		
Primary school	8	8.0
Middle School	26	26.0
High school	38	38.0
University	28	28.0
Patient's relative marital status		
Married	78	78.0
Single	22	22.0
Number of children of the patient's relative		
Has no children	22	22.0
1–2 children	38	38.0
3–4 children	35	35.0
5 children and more	5	5.0
Degree of relationship		
Daughter	42	42.0
Son	27	27.0
Husband/wife	11	11.0
Sister/brother	15	15.0
Mother	2	2.0
Father	1	1.0
Grandson	2	2.0
Patient's relative’s history of previous ICU visit		
Yes	49	49.0
No	51	51.0
Patient’s relative’s history of previous psychiatric treatment		
Yes	18	18.0
No	82	82.0
Living in the same house with the patient		
Yes	37	37.0
No	63	63.0

**Table 2:** Sociodemographic characteristics of inpatients in the intensive care unit

	Frequency (n)	Percentage (%)
Gender		
Female	54	54.0
Male	46	46.0
Diagnosis		
Cardiovascular	22	22.0
Respiratory	15	15.0
Neurological	20	20.0
Digestive	2	2.0
Metabolic	6	6.0
Oncological	30	30.0
Renal	3	3.0
Post-operative complications	2	2.0

	Me. (S.D.)	(Min. – Max.)
Patient age	70.15 (14.88)	20–96
Length of stay	17.96 (14.53)	3–69
GCS	6.89 (4.58)	3–15
APACHE II score	75.95 (12.59)	21–97

Me., mean; Min., minimum; Max., maximum; S.D., standard deviation; GCS, Glasgow coma score; APACHE II, Acute Physiology and Chronic Health Evaluation II

**Table 3:** Emotional reaction distributions of patient relatives

	Frequency (n)	Percentage (%)
Normal perception	55	55.0
Initial shock reaction	14	14.0
Denial	8	8.0
Feeling guilty	19	19.0
Anger	4	4.0

The relationships between the sociodemographic characteristics of inpatients in the ICU and their relatives and emotional reactions to the bad news are given in Table 4. In this table, there was a significant positive correlation between ***normal response*** levels and PR age (r = 0.287, *P* < 0.01), PR gender (r = 0.246, *P* < 0.05), the number of children of PR(r = 0.354, *P* < 0.01), PR previous psychiatric treatment history (r = 0.361, *P* < 0.01), living in the same house as the patient (r = 0.264, *P* < 0.01), length of stay (r = 0.404, *P* < 0.01), patient age (r = . 663, *P* < 0.01), and APACHE II score (r = 0.258, *P* < 0.01). However, a negative significant correlation was found between normal response levels and PR marital status (r = − 0.393, *P* < 0.01), and PR previous ICU visit history (r = − 0.243, *P* < 0.05).

**Table 4:** Correlation matrix results showing the relationship between the variables belonging to the participants

	A:	B:	C:	D:	E:	F:	G:	H:	I:	J:	K:	L:	M:	N:	O:	P:	Q:	R:	S:	T:
A: Normal reaction	–																			
B: Initial shock reaction	− .446**	–																		
C: Denial	− .326**	− .119	–																	
D: Feeling guilty	− .518**	− .189	− .138	–																
E: Anger	− .226*	− .082	− .060	− .096	–															
F: Patient’s relative age	.287**	.085	− .062	− .329**	− .188	–														
G: Patient’s relative gender	.246*	− .153	− .015	− .170	− .063	.099	–													
H: Education level of patient’s relatives	− .043	− .100	.027	.135	− .020	− .361**	.178	–												
I: Patient's relative marital status	− .393**	.064	.199*	.191	.261**	− .460**	− .089	.251*	–											
J: Number of children of the patient's relative	.354**	− .029	− .044	− .273**	− .236*	.619**	.021	− .537**	− .648**	–										
K: Degree of relationship	− .102	.141	.224*	− .114	− .152	.394**	.424**	− .124	− .137	.300**	–									
L: Patient's relative’s history of previous ICU visit	− .243*	.107	− .080	.199*	.200*	− .127	− .220*	− .119	.183	− .200*	− 0.180	–								
M: Patient’s relative’s history of previous psychiatric treatment	.361**	− .411**	.042	.084	− .436**	.201*	.117	− .034	− .191	.222*	0.016	.009	–							
N: Living in the same house with the patient	.264**	− .109	.073	− .180	− .161	.168	.076	− .002	− .493**	.353**	− .077	− .047	.234*	–						
O: Length of stay in İCU	.404**	− .160	− .149	− .199*	− .180	.168	.042	− .099	− .141	.214*	.115	− .020	.150	.096	–					
P: Diagnosis	.140	.043	− .150	− .096	− .092	.021	.111	.065	− .193	.032	− .010	− .307**	− .009	− .039	− .006	–				
Q: Patient's age	.663**	− .254*	− .267**	− .339**	− .113	.361**	.050	− .106	− .296**	.249*	− .345**	.015	.249*	.299**	.315**	− .048	–			
R: Patient's gender	− .093	.032	− .050	.090	.119	− .139	− .139	− .118	− .103	.046	− .018	.102	− .090	− .082	− .094	− .038	− .130	–		
S: GCS	− .032	.128	.174	− .171	− .082	.026	.300**	.087	.111	− .020	.212*	− .366**	− .111	− .028	− .005	.222*	− .138	− .035	–	
T: APACHE II score	.258**	.068	− .192	− .221*	− .019	.172	.182	.094	− .249*	.191	− .084	− .210*	.056	.068	− .131	.141	.199*	− .005	− 0.124	–

ICU, intensive care unit; GCS, Glasgow coma scale; APACHE II, Acute Physiology and Chronic Health Evaluation II

^*^P < .05, **P < .01

There was a significant negative correlation between the ***initial shock response*** and PR previous psychiatric treatment history (r = − 0.441, *P* < 0.01) and patient age (r = − 0.254, *P* < 0.05).

There was a significant positive correlation between ***denial response*** and PR marital status (r = 0.199, p < 0.01), and degree of closeness (r = 0.224, *P* < 0.05). However, a negative significant correlation was found between denial response and patient age (r = − 0.267, *P* < 0.01).

There was a significant positive correlation between PR ***feelings of guilt*** and PR previous ICU visit history (r = 0.199,* P* < 0.05). However, a negative significant correlation was found between PR feelings of guilt and PR age (r = − 0.329, *P* < 0.01), number of children of PR (r = − 0.273, *P* < 0.05), length of stay (r = − 0.199, *P* < 0.05), patient age (r = − 0.399, *P* < 0.01), and APACHE II scores (r = − 0.221, *P* < 0.01).

There was a significant positive correlation between ***anger response*** and PR marital status (r = 0.261, *P* < 0.01), and PR previous ICU visit history (r = 0.200, *P* < 0.05). However, a negative significant correlation was found between anger response and number of children of PR (r = − 0.236, *P* < 0.05), and PR previous psychiatric treatment history (r = − 0.436, *P* < 0.01).

The emotional grief reactions of the relatives were divided into five categories, and MNLR analysis was performed to predict which variables affected the categories.

The results of the MNLR analysis performed to determine to what extent each independent variable (PR gender, PR age, PR education level, PR marital status, number of children of PR, degree of relationship, PR history of previous ICU visit, PR history of previous psychiatric treatment, living in the same house as the patient, length of stay in ICU, diagnosis, patient age, patient gender, GCS, APACHE II score) predicted the dependent variable (emotional reactions) are given in Table 5 and shows comparisons between the emotional response status of the patient’s relatives with the reference category (normal perception).

**Table 5:** Multinominal logistic regression analysis results according to emotional response level^a^

	Independent variables	B	SE	OR	*P* value
Initial shock reaction	Constant	− 1824.7	171,860.6	–	.992
	Patient's relative gender	− 971.4	51,835.6	0	.985
	Patient's relative age	13.9	820.9	1,121,623.4	.986
	Education level of the patient’s relatives	− 404.0	48,500.8	3.39E−176	.993
	Patient's relative marital status	− 646.7	59,258.7	1.40E−281	.991
	Number of children of the patient's relative	− 1016.0	57,912.6	0	.986
	Degree of relationship	159.3	8912.7	1.52E+69	.986
	Patient's relative’s history of previous ICU visit	1197.7	69,896.8	^b^	.986
	Patient’s relative’s history of previous psychiatric treatment	− 1351.5	74,962.5	0	.986
	Living in the same house with the patient	610.9	32,989.2	2.01E+265	.985
	Length of stay in İCU	13.2	798.4	527,834.475	.987
	Diagnosis	137.5	8960.0	5.34E+59	.988
	Patient age	− 15.7	1104.7	1.57E−07	.989
	Patient gender	92.5	27,804.1	1.47E+40	.997
	GCS	139.9	7629.5	5.64E+60	.985
	APACHE II score	42.2	2500.5	2.19015E+18	.987
Denial	Constant	− 3.12	59.7		.958
	Patient's relative gender	− 2.41	2.15	0.09	.261
	Patient's relative age	0.013	0.10	1.01	.899
	Education level of the patient’s relatives	− 1.17	0.96	0.31	.224
	Patient's relative marital status	7.34	4.87	1543.3	.131
	Number of children of the patient's relative	− 2.07	1.87	0.13	.269
	Degree of relationship	1.63	1.11	5.08	.141
	Patient's relative’s history of previous ICU visit	− 0.22	2.11	0.80	.918
	Patient’s relative’s history of previous psychiatric treatment	3.28	29.2	26.6	.911
	Living in the same house with the patient	7.03	3.95	1133.2	.075
	Length of stay in İCU	− 0.18	0.14	0.83	.189
	Diagnosis	− 0.36	0.44	0.7	.413
	Patient age	− 0.29	0.13	0.75	.020*
	Patient gender	− 1.70	2.16	0.18	.430
	GCS	− 0.07	0.23	0.93	.752
	APACHE II score	0.07	0.10	1.07	.485
Feeling guilty	Constant	17.2	7.78		.027
	Patient's relative gender	0.31	1.79	1.37	.862
	Patient's relative age	0.16	0.12	1.17	.193
	Education level of the patient’s relatives	0.11	0.68	1.12	.872
	Patient's relative marital status	3.09	2.52	22.15	.220
	Number of children of the patient's relative	− 1.73	1.17	0.177	.140
	Degree of relationship	− 1.45	1.54	0.23	.345
	Patient's relative’s history of previous ICU visit	1.50	1.38	4.499	.275
	Patient’s relative’s history of previous psychiatric treatment	− 1.36	2.02	0.26	.501
	Living in the same house with the patient	2.31	2.09	10.1	.269
	Length of stay in İCU	− 0.03	0.03	0.97	.294
	Diagnosis	− 0.19	0.29	0.82	.500
	Patient age	− 0.36	0.15	0.698	.018*
	Patient gender	0.12	1.27	1.13	.926
	GCS	− 0.18	0.17	0.83	.290
	APACHE II score	− 0.024	0.05	0.98	.645
Anger	Constant	− 1704.8	0.00		–
	Patient's relative gender	− 846.4	0.00	0	
	Patient's relative age	14.44	2642.9	1,868,664.2	.996
	Education level of the patient’s relatives	− 403.3	40,658.4	6.97E−176	.992
	Patient's relative marital status	− 619.5	0.000	9.38E−270	
	Number of children of the patient's relative	− 971.96	0.000	0	
	Degree of relationship	130.8	26,488.5	6.17E+56	.996
	Patient's relative’s history of previous ICU visit	1273.6	103,748.5	^b^	.990
	Patient’s relative’s history of previous psychiatric treatment	− 1388.7	0.000	0	
	Living in the same house with the patient	569.5	65,826.9	2.24E+247	.993
	Length of stay in ICU	12.92	3519.4	406,812.761	.997
	Diagnosis	139.97	16,217.5	6.16E+60	.993
	Patient age	− 14.99	1863.02	3.09E−07	.994
	Patient gender	94.5	38,342.18	1.14E+41	.998
	GCS	129.68	6065.2	2.08E+56	.983
	APACHE II score	37.71	2719.8	2.37153E+16	.989

SE, standard error; OR, odds ratio; GCS, Glasgow coma scale; ICU, intensive care unit

^a^The reference category is: Normal reception (p < 0.05); ^b^This parameter is set to zero because it is redundant

^*^If the *P* value was < 0.05, it was considered significant

In this study, according to the model fit information estimation results, the model as a whole was found to be significant [χ^2^ (60) = 201.240, *P* < 001]. The goodness-of-fit table contains the deviation and Pearson chi-square tests, which are useful in determining whether a model fits the data well. Non-significant test results indicate that the model fits the data well [10]. Pearson’s chi-square test [χ^2^ (336) = 48.838, *P* = 1.00] and deviance chi-square test [χ^2^ (336) = 59.660, *P* = 1.00] showed that the model fit the data well. The pseudo R2 values of the model were R2 = 0.866 (Cox and Snell), R2 = 0.944 (Nagelkerke) and R2 = 0.805 (McFadden).

The first set of coefficients in the MNLR analysis in Table 5 represents the comparisons between the normal responders and the initial shock responders. All of the independent variables in the model do not explain the emotional changes in the initial shock responders compared to the normal responders (*P* > 0.05).

The second set of coefficients represents comparisons between the normal responders and the denial responders. Only “patient age” was a significant determinant in the model (B = − 0.293, S.E. = 0.126, *P* = 0.020), because relatives of patients who were older were less likely to give a denial response than a normal response. The probability ratio of 746 shows that for a one-unit increase in the patient’s age, the probability of denial among relatives of the patient decreased by 746 times.

The third set of coefficients represents the comparisons between the normal responders and those who feel guilty. Only “patient age” was a significant determinant in the model (B = − 0.360, S.E. = 0.153, *P* = 0.018), because relatives of patients who were older were less likely to have a guilty reaction than a normal response. The probability ratio of 698 shows that for one unit increase in the patient’s age, the probability of feeling guilty by the relatives of the patient decreased by 698 times.

The last set of coefficients represents the comparisons between the normal responders and those who react with anger. All of the independent variables in the model do not explain the emotional changes in the anger responders compared to the normal responders (*P* > 0.05).

## Discussion

Death, which is the inevitable end for every human, always leaves grieving people behind. Grief is a natural response to the death of a loved one [11]. The grief response is unique and differs from person to person. This reaction may vary depending on the deceased person and some factors related to the person who lost their loved one. It is described as one of the most distressing life experiences. People may have close relationships with many people to varying degrees throughout their lives. There is no standard definition of grief reaction that determines who should mourn after a death [12]. Relatives and close friends are known to be most affected by a person’s death. These include at least one person most likely to react to death. In our study, the person most likely to react to death, who visited the patient most frequently in the ICU, regularly received information, and was the legal heir of the patient, was determined and included in the study.

The onset of grief may vary depending on whether the death occurs in a short or long period of time. In fact, grief begins with the realization that the loved one is going to die [13]. Therefore, in our study, patients with an expected death rate of more than 50%, calculated with the APACHE II score on the first day of admission to the ICU, were included in the study and this was explained to the relatives of the patients. Thus, in the minds of the relatives, the idea that their family member can die at any time was formed and the grief process initiated in a way from the first day of admission to the ICU. Grief reactions to the news of sudden death were eliminated by including patients whose hospitalization period was longer than three days in this study.

Studies show that initial reactions to the news of death (such as rejection, denial, or guilt) can guide the estimation of the possibility of a prolonged grieving process and progression to traumatic or pathological grief [14, 15]. Early detection and differentiation of risky groups in terms of traumatic grief and follow-up can provide earlier and more specific treatment. In such cases, better prognosis, faster recovery of functionality, and an earlier and uncomplicated return to normal life can be achieved. It has been reported that 40–60% of PRs experiencing pathological grief have major depression and suicidal ideation, and suicidal behavior is observed in some of these individuals [16]. For PRs, it is important that the predisposing factors of traumatic grief are well known by health care professionals in order to avoid such undesired results. Thus, preventive health care services and support services for individuals at risk can be planned in advance and the development of traumatic grief can be prevented.

In the study, the possible grief reactions of the PR at the time of death were evaluated in five categories; normal, first shock reaction, denial, guilt and anger. The majority of PR (55%) accepted the news of death as normal. The normal acceptance rate was found to be statistically significantly higher in people who had children, did not have a history of psychiatric treatment, did not share the same house as the patient, were older, and were female. Factors affecting the PR’s normal acceptance of the news of death were determined as the patient’s advanced age, long hospitalization period, and poor general health status (high APACHE II score) at the time of admission to the ICU. However, normal response behavior was found to be statistically significantly lower in people who were married and who had not visited the ICU because of another relative before. Bolton et al. [17] found that people who grieve after sudden and unexpected death are three times more likely to have psychological disorders such as depression or anxiety than those who grieve after a natural death. In our study, the normal response rate to the news of death was found to be high, as the long-term ICU hospitalization of the patients who died, their older ages, and their high APACHE II scores kept the PRs from experiencing sudden and unexpected death. In a meta-analysis, it was determined that when one member of a married couple sharing the same house dies, 22% of the survivors experience major depression in the first year during the grieving process [18]. In line with the findings of that meta-analysis, in our study, it was found that the probability of responding normally to the news of death was low among married people and high among those who did not share a house with the deceased. It was predictable that those who had children went through this process more healthily and showed a normal reaction to the news of death due to not feeling alone during the grieving process and sharing love with their children. In a survey conducted by Kersting et al. [19] with 2520 people, it was determined that pathological grieving processes were more common among women, those with a history of psychiatric treatment, and older people (> 61 years). In line with this, in our study, the rate of responding normally to loss (i.e., news of death) was found to be higher among those who did not have a history of psychiatric treatment. However, among elderly and female PRs, a higher rate of normal response to loss was observed. The mean age of PRs in our study was 48.7 years and 60% were women. We think the fact that our sample group was younger and smaller led to these differences in findings.

According to the results of our study, 14% of the PRs showed an initial shock reaction to the news of death. Statistically lower rate of initial shock reaction was seen in people who did not have previous psychiatric treatment history and in patients with advanced age. The denial response rate was 8%. The parents of 69% of the participants in the study were not living. Statistically significantly higher rates of denial reaction were seen in married people and those whose parents had died. However, the most important cause of the denial response was the age of the patient when compared to the normal responders, and the probability of the PR to show a denial response increased (746 times) as the age of the patient decreased.

In the study, a guilt reaction was observed at a rate of 19%. It was statistically determined that guilt was more common in the PRs who had not visited the ICU for any other relatives before, but the feeling of guilt was less common in the PRs who were older and had children. At the same time, the sense of guilt was also statistically less in the relatives of the patients who were older and had a high APACHE-2 score. When the normal responders and those who felt guilty were compared, the most important determinant was the age of the patient. As the age of the deceased patient decreased the rate of feeling guilty increased 698 times. In a study conducted by Stroebe et al. [20], it was observed that self-blame after the loss of a loved one is a strong predictor of a distressing grieving process. Lobb et al. [21] also reported the risk of pathological grief among those who have not gone through a similar experience before, those who were unprepared for the death, and those who feel lonely in life in a systematic review of the literature, supporting the findings of the present study. In the study, the rate of PRs whose children died was 3%. In a study, it was reported that the probability of traumatic and long-lasting grief increased up to 60%, especially in those who lost their young children [22]. In cases where the deceased is young, the death is considered unusual due to being untimely. It has been reported in many studies that intense emotional feelings such as denial, rejection, and guilt develop in these cases of unusual deaths at younger ages and long-lasting traumatic grieving processes are observed [21, 23–25]. Similarly, in our study, as the ages of the patients who died decreased, the rates of denial and guilt about the loss increased among PRs.

In the present study, the least common grief reaction was anger with a rate of 4%. However, in this study, no difference was found between those who responded normally to the news of death and those who demonstrated reactions of anger in terms of independent variables in the study model. We attribute this to the fact that anger is a basic emotion and a behavioral pattern mostly related to personality structure. The expression of anger varies from person to person. Some people have adapted to acting with anger as a lifestyle and a personality trait. Angry behavior is frequently observed in people with certain personality types, such as borderline, narcissistic, or antisocial personalities [26, 27].

Reactions of the PRs to the news of death often involve intense sadness. The health care providers must be ready to deal with various emotional outbursts. The news of sudden and unexpected death is often given in the emergency departments and ICUs of hospitals. Announcing the death of a patient they are treating is one of the most stressful moments for any physician. The main purpose of medical education is to restore health, resuscitate and cure the patient. Most medical schools do not have a required course or program on death notifications. For this reason, physicians may feel helpless and uncomfortable because they cannot decide how to approach the grieving individual. In a study by Benenson et al. [7] about emergency medicine residents, 95% of residents who received training about death notification achieved satisfactory results. Hobgood et al. [28, 29] developed the GRIEV_ING mnemonic model and showed that by training the emergency service personnel with this model, they could improve their confidence and competence in death notification. Physicians can overcome this difficult task with such models and training. It is very important to increase the number of physicians who receive such training in order not to complicate the grief process of the person receiving notification of death.

Our study has some limitations. One of them is that the death notification was made not by a single physician but by three different physicians. Evaluations by three different physicians may have caused different interpretations of grief reactions. The second is that the study was conducted only in the adult ICU. This situation caused great differences in the distribution of patient—PR (69% parents, 3% children).

In conclusion, the denial and guilt rate in the grief reactions of PRs when they receive the news of the death of their loved ones in the ICU increases as the age of the deceased gets younger. In this case, PRs may need special attention. Although grief is expressed as a natural reaction after a loss, it can turn into a long-term, complicated mourning response if it is not managed well by healthcare professionals. For this reason, physicians should improve their communication and be more careful in their behaviors and attitudes towards PRs, especially during the death notification process of young patients.

## Data Availability

The datasets generated and/or analyzed during the current study are not publicly available due to the Personal Data Protection Law enacted in 2016 in Turkey but are available from the corresponding author (barguv@gmail.com) on reasonable request.

## References

[1] Özel Y, Özkan B (2020). Psychosocial approach to loss and mourning. Psikiyatride Guncel Yaklasimlar. Current Approaches Psychiatry.

[2] Brown-Saltzman K. Transforming the grief experience In. Carroll Johnson RM, Gorman LM, Bush NJ, editors. Oncology Nursing Society. 2nd edn.; 2006. p. 293–312.

[3] Tyrrell PSH, Siddiqui W. Stages of dying. StatPearls Publishing. https://www.ncbi.nlm.nih.gov/books/NBK507885/. Accessed 6 Apr 2021. 29939662

[4] White P, Ferszt G (2009). Exploration of nurse practitioner practice with clients who are grieving. J Am Acad Nurse Pract.

[5] Morris SE, Nayak MM, Block SD (2020). Insights from bereaved family members about end-of-life care and bereavement. J Palliat Med.

[6] Galazzi A, Adamini I, Bazzano G, Cancelli L, Fridh I, Laquintana D (2021). Intensive care unit diaries to help bereaved family members in their grieving process: a systematic review. Intensive Crit Care Nurs.

[7] Benenson RS, Pollack ML (2003). Evaluation of emergency medicine resident death notification skills by direct observation. Acad Emerg Med.

[8] Statistics Canada. Table 13-10-0715-01 Deaths, by place of death (hospital or non-hospital). 10.25318/1310071501-eng.

[9] Naik SB (2013). Death in the hospital: breaking the bad news to the bereaved family. Indian J Crit Care Med.

[10] Petrucci CJ (2009). A primer for social worker researchers on how to conduct a multinomial logistic regression. J Soc Serv Res.

[11] Zisook S, Simon NM, Reynolds CF, Pies R, Lebowitz B, Young IT (2010). Bereavement, complicated grief, and DSM, part 2: complicated grief. J Clin Psychiatry.

[12] Antonucci T, Akiyama H, Takahashi K (2004). Attachment and close relationships across the life span. Attach Hum Dev.

[13] A lifecare guide to grief and bereavement. LifeCare, Inc. 2001. https://dokumen.tips/documents/lifecare-guide-to-grief-and-bereavement.html Accessed 20 May 2021.

[14] Simon NM, Wall MM, Keshaviah A, Dryman MT, LeBlanc NJ, Shear MK (2011). Informing the symptom profile of complicated grief. Depress Anxiety.

[15] Çelik FGH, Hocaoğlu Ç (2015). After landslide in Rize 'traumatic grief': three case studies. J Clin Psychiatry.

[16] Madeira L, Miranda AT (2021). A narrative review of suicide: aiming at a more encompassing understanding. Philosophies.

[17] Bolton JM, Au W, Leslie WD, Martens PJ, Enns MW, Roos LL (2013). Parents bereaved by offspring suicide: a population-based longitudinal case-control study. JAMA Psychiat.

[18] Onrust SA, Cuijpers P (2006). Mood and anxiety disorders in widowhood: a systematic review. Aging Ment Health.

[19] Kersting A, Brähler E, Glaesmer H, Wagner B (2011). Prevalence of complicated grief in a representative population-based sample. J Affect Disord.

[20] Stroebe M, Stroebe W, Van de Schoot R, Schut H, Abakoumkin G, Li J (2014). Guilt in bereavement: the role of self-blame and regret in coping with loss. PLoS ONE.

[21] Lobb EA, Kristjanson LJ, Aoun SM, Monterosso L, Halkett GK, Davies A (2010). Predictors of complicated grief: a systematic review of empirical studies. Death Stud.

[22] Meert KL, Shear K, Newth CJL, Harrison R, Berger J, Zimmerman J (2011). Follow-up study of complicated grief among parents eighteen months after a child’s death in the pediatric intensive care unit. J Palliat Med.

[23] Chachar AS, Younus S, Ali W (2021). Developmental understanding of death and grief among children during COVID-19 pandemic: application of Bronfenbrenner's bioecological model. Front Psychiatry.

[24] Boelen PA, Smid GE (2017). Disturbed grief: prolonged grief disorder and persistent complex bereavement disorder. BMJ.

[25] Prigerson HG, Boelen PA, Xu J, Smith KV, Maciejewski PK (2021). Validation of the new DSM-5-TR criteria for prolonged grief disorder and the PG-13-Revised (PG-13-R) scale. World Psychiatry.

[26] Zanarini MC, Frankenburg FR, Reich DB, Fitzmaurice GM (2016). Fluidity of the subsyndromal phenomenology of borderline personality disorder over 16 years of prospective follow-up. Am J Psychiatry.

[27] Williams R (2017). Anger as a basic emotion and its role in personality building and pathological growth: the neuroscientific, developmental and clinical perspectives. Front Psychol.

[28] Hobgood C, Harward D, Newton K, Davis W (2005). The educational intervention “GRIEV_ING” improves the death notification skills of residents. Acad Emerg Med.

[29] Hobgood C, Mathew D, Woodyard DJ, Shofer FS, Brice JH (2013). Death in the field: teaching paramedics to deliver effective death notifications using the educational intervention “GRIEV_ING”. Prehospital Emerg Care.

